# Left Ventricular Ejection Fraction in Patients With Ovarian Cancer Treated With Avelumab, Pegylated Liposomal Doxorubicin, or Both

**DOI:** 10.1093/oncolo/oyad213

**Published:** 2023-09-04

**Authors:** Marc P Bonaca, Javid J Moslehi, Jonathan A Ledermann, Elisabete Michelon, Caimiao Wei, Michael Moran, Bradley J Monk, Eric Pujade-Lauraine

**Affiliations:** Colorado Prevention Center Clinical Research, Division of Cardiology, Department of Medicine, University of Colorado School of Medicine, Aurora, CO, USA; Section of Cardio-Oncology and Immunology, Division of Cardiology and the Cardiovascular Research Institute, UCSF School of Medicine, Cardiovascular Research Institute (CVRI), San Francisco, CA, USA; UCL Cancer Institute and UCL Hospitals, London, UK; Pfizer, New York, NY, USA; Pfizer, Groton, CT, USA; Pfizer Pharma GmbH, Berlin, Germany (Affiliation at the time the research was conducted); Arizona Oncology (US Oncology Network), University of Arizona and Creighton University, Phoenix, AZ, USA; ARCAGY-GINECO, Paris, France

**Keywords:** clinical trial, phase III, immune checkpoint inhibitors, immunotherapy, ventricular function, left, carcinoma, ovarian epithelial

## Abstract

In the phase III JAVELIN Ovarian 200 trial, 566 patients with platinum-resistant/refractory ovarian cancer were randomized 1:1:1 to receive avelumab alone, avelumab plus pegylated liposomal doxorubicin (PLD), or PLD alone. Cardiac monitoring was included for all patients. We report left ventricular ejection fraction (LVEF) data from the trial. Grade ≥3 cardiac adverse events (AEs) occurred in 4 (2.1%), 1 (0.5%), and 0 patients in the avelumab, combination, and PLD arms, respectively. LVEF decreases of ≥10% to below institutional lower limit of normal at any time during treatment were observed in 1 (0.8%), 3 (1.9%), and 2 (1.5%) patients, respectively; 4 had subsequent assessments, and these showed transient decreases. No patient had a cardiovascular AE related to LVEF decrease. This analysis is, to our knowledge, the first analysis of LVEF in patients receiving immune checkpoint inhibitors.

**ClinicalTrials.gov Identifier:**

NCT02580058.

## Teaser

In the phase III JAVELIN Ovarian 200 trial, 566 patients with platinum-resistant/refractory ovarian cancer were randomized 1:1:1 to receive avelumab alone, avelumab plus pegylated liposomal doxorubicin (PLD), or PLD alone. Cardiac monitoring was included for all patients. This article reports left ventricular ejection fraction data from the trial.

## Background

Immune checkpoint inhibitors (ICIs) have revolutionized anticancer treatment and are increasingly being combined with chemotherapies or targeted therapies to enhance antitumor activity. ICIs are associated with immune-related cardiovascular adverse events (AEs), including myocarditis.^1^ In addition, reports of noninflammatory cardiomyopathy, albeit from retrospective studies, suggest that ICI-induced toxicities may lead to cardiac dysfunction potentially through direct mechanisms.^2^ Few studies of ICIs have prospectively and systematically collected left ventricular ejection fraction (LVEF) data using echocardiography or multigated acquisition (MUGA) scans. Avelumab, an anti-programmed death ligand 1 ICI, is an approved treatment for several tumors.^3,4^ In the phase III JAVELIN Ovarian 200 trial, patients with platinum-resistant or platinum-refractory ovarian cancer were randomized to receive avelumab alone, avelumab plus pegylated liposomal doxorubicin (PLD), or PLD alone.^5^ The trial failed to meet its primary endpoints of improved progression-free survival and overall survival with avelumab alone or combined with PLD vs. PLD alone.^5^ No new safety signals were observed. Because PLD is an anthracycline associated with cardiotoxicity,^6^ LVEF was assessed in each patient using systemic and sequential cardiac monitoring. Here, we report LVEF data from the trial.

## Methods

The design of the multicenter, open-label, phase III JAVELIN Ovarian 200 trial (NCT02580058) has been described previously.^5^ Briefly, eligible women had advanced ­platinum-resistant or platinum-refractory epithelial ovarian, fallopian tube, or peritoneal cancer and had received 1-3 prior treatment lines for platinum-sensitive disease and none for ­platinum-resistant disease. Exclusion criteria included baseline LVEF <50% (by echocardiography or MUGA scans) and prior ­anthracycline-related cardiotoxicity or ­anthracycline exposure approaching the lifetime limit (450-550 mg/m^2^). Full eligibility criteria have been reported previously.^5^ Patients were randomized 1:1:1 to receive avelumab 10 mg/kg intravenously every 2 weeks, PLD 40 mg/m^2^ intravenously every 4 weeks, or avelumab plus PLD. Cardiac monitoring was performed throughout the trial, and comprised electrocardiogram at baseline and every 4 weeks and LVEF assessment by MUGA scans or echocardiography at baseline and every 8 weeks until discontinuation or end of treatment. Electrocardiogram and LVEF results were read locally. Decrease in LVEF to below institutional lower limit of normal (LLN) or by ≥15% from baseline required treatment interruption, and LVEF was reassessed within 45 days and every 3 months. Upon recovery (LVEF increase to within 5% of baseline within 45 days from nadir), treatment could be resumed at investigator’s discretion, and an additional LVEF evaluation was performed within 2 weeks. AEs were investigator reported, analyzed descriptively, and graded using National Cancer Institute Common Terminology Criteria for Adverse Events, version 4.03.

## Results

Overall, 566 patients were randomized (avelumab, *n* = 188; avelumab plus PLD, *n* = 188; PLD, *n* = 190). At data cutoff (September 19, 2018), median duration of treatment was 10.1 weeks (interquartile range [IQR], 7.0-19.4) in the avelumab alone arm, 16.9 weeks (IQR, 9.1-35.9) for avelumab and 16.3 weeks (IQR, 8.1-32.0) for PLD in the avelumab plus PLD arm, and 16.0 weeks (IQR, 8.0-25.0) in the PLD alone arm.

Demographic characteristics were similar between arms.^5^ Median age was 60 years, and most patients were never smokers (73.5%). At screening, 40 patients (7.1%) had diabetes mellitus, 79 patients (14.0%) had hypercholesterolemia, and 185 patients (32.7%) had hypertension, with broadly similar proportions between arms. One patient (0.5%) in the avelumab alone arm reported ongoing heart failure at screening.

Among patients who received ≥1 dose of study treatment in each arm (avelumab, *n* = 187; avelumab plus PLD, *n* = 182; PLD, *n* = 177), similar proportions received cardiovascular concomitant therapies (started before or during study treatment), including β-blockers (*n* = 71; 13.0%), calcium-channel blockers (*n* = 71; 13.0%), agents targeting the ­renin-angiotensin system (*n* = 86; 15.8%), and lipid-modifying agents (*n* = 104; 19.0%). During treatment, AEs reported under the Medical Dictionary for Regulatory Activities system organ class of cardiac disorders of any grade occurred in 15 patients (8.0%) in the avelumab alone arm, 20 patients (11.0%) in the avelumab plus PLD arm, and 13 patients (7.3%) in the PLD alone arm, and those of grade ≥3 occurred in 4 patients (2.1%; atrial fibrillation, atrial flutter, sinus tachycardia, and cardiac failure [*n* = 1 each]), 1 patient (0.5%; myocardial infarction [*n* = 1]), and 0 patients, respectively. Myocarditis was not reported by any patient during this study.

**Figure 1.**
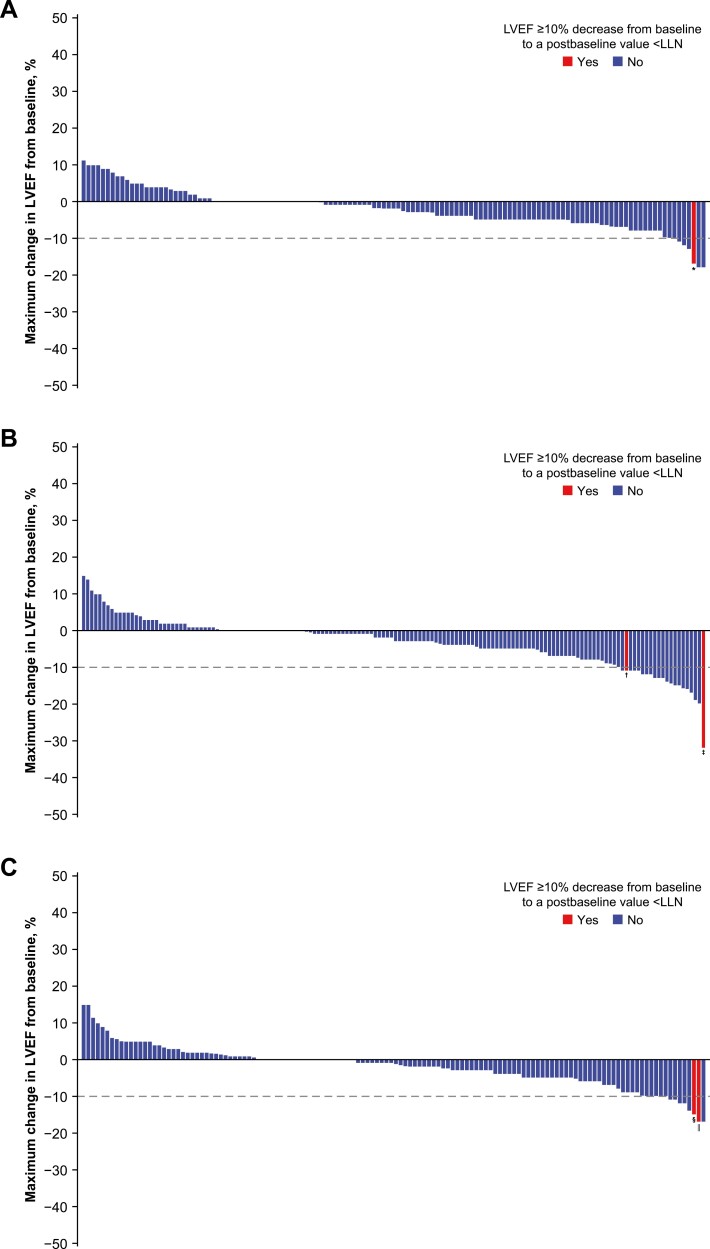
Maximum change in LVEF from baseline during treatment in the (**A**) avelumab alone, (**B**) avelumab plus PLD, and (**C**) PLD alone arms. One patient in the avelumab plus PLD arm (panel B) had an LVEF decrease of 11% that was <LLN at time of assessment; however, their maximum LVEF decrease of 13% (included in the figure) was not <LLN. *Decrease from 63% to 46%; patient subsequently recovered. ^†^Decrease from 54% to 43%; no subsequent assessments. ^‡^Decrease from 68% to 36%; patient subsequently recovered. ^§^Decrease from 64% to 49%; patient subsequently recovered. ^‖^Decrease from 68% to 51%; patient subsequently recovered. Abbreviations: LLN: lower limit of normal; LVEF: left ventricular ejection fraction; PLD: pegylated liposomal doxorubicin.

At baseline, 1 patient (avelumab plus PLD arm) had an LVEF <LLN. Figure 1 shows maximum LVEF change from baseline during treatment in patients with baseline and postbaseline on-treatment LVEF assessments (*n* = 415 [avelumab, *n* = 129; avelumab plus PLD, *n* = 154; PLD, *n* = 132]). During follow-up, LVEF decreases of ≥10% to <LLN at any time during the on-treatment period were rare and were only observed in 1 patient (0.8%) in the avelumab alone arm, 3 patients (1.9%) in the avelumab plus PLD arm, and 2 patients (1.5%) in the PLD alone arm. Of these 6 patients, 2 did not have subsequent assessments (treatment was discontinued due to disease progression; both in the avelumab plus PLD arm). For the remaining 4 patients with subsequent assessments, LVEF decreases were transient, and recovery occurred without therapeutic intervention. One patient in the avelumab plus PLD arm had an LVEF decrease from 68% to 36% and subsequently recovered to >LLN (50%) within 1 month. None of these 6 patients had a cardiovascular AE (ie, symptomatic heart failure) related to LVEF decrease.

## Discussion

JAVELIN Ovarian 200 was an open-label, phase III trial that was designed to include cardiac monitoring for LVEF in all patients across all 3 arms due to the administration of PLD.^5^ To our knowledge, this is the first and largest analysis of LVEF in patients receiving ICIs and the largest analysis in patients with ovarian cancer receiving PLD. In patients with preserved LVEF at baseline, significant LVEF decreases were rare with avelumab or PLD treatment alone or in combination. Observed changes in LVEF were transitory and were not associated with cardiovascular AEs. These findings align with the absence of increased cardiomyopathy or heart failure seen in other avelumab studies^7,8^ and the relatively low rate of cardiotoxicity with PLD treatment in ovarian cancer.^6^

## Data Availability

Upon request, and subject to review, Pfizer will provide the data that support the findings of this study. Subject to certain criteria, conditions, and exceptions, Pfizer may also provide access to the related individual deidentified participant data. See https://www.pfizer.com/science/clinical-trials/trial-data-and-results for more information.
